# Serious adverse events and compensation in registration trials: a review of data from a Japanese university hospital

**DOI:** 10.1186/1756-0500-7-245

**Published:** 2014-04-17

**Authors:** Miho Watanabe, Soichiro Tajima, Rumi Katashima, Toshiko Miyamoto, Makiko Yamagami, Kazuo Minakuchi, Hiroaki Yanagawa

**Affiliations:** 1 Clinical Trial Center for Developmental Therapeutics, Tokushima University Hospital, Kuramoto-cho 2, Tokushima 770-8503, Japan; 2 Institutional Review Board, Tokushima University Hospital, Kuramoto-cho, Tokushima, Japan

**Keywords:** Registration trial, Serious adverse events, Compensation, Clinical research coordinator, Oncology

## Abstract

**Background:**

Clinical trials leading to regulatory approval, or registration trials, play a central role in the development of drugs and medical devices. The contribution of support staff, such as the clinical research coordinator (CRC) and administrative officers, in registration trials is now widely recognized. Attending to serious adverse events is an important duty of the CRC and investigators alike, and managing these complications and compensation constitutes a key responsibility. We retrospectively examined the frequency of serious adverse events and compensation events reported from 2007 through 2011 at Tokushima University Hospital, an academic hospital in rural Japan. We present herein the results of our analysis.

**Results:**

Over the five-year period, 284 subjects participating in 106 registration trials experienced a total of 43 serious adverse events, and eight compensation events were documented. Among the serious adverse events, 35 (81.4%) were considered not related to the investigational drug, and 17 (39.5%) resulted in withdrawal of the study drug. Patients with malignant diseases experienced serious adverse events significantly more frequently compared to those with non-malignant diseases (28.3% versus 8.2%, respectively; *P* < 0.01).

**Conclusions:**

The CRC should be vigilant for serious adverse events in oncology clinical trials due to the generally higher frequency of these complications in subjects with malignancy. However, on an individual basis, the CRC may be seldom involved in the process for compensating serious adverse events. Therefore, the CRC’s ability to share such experiences may serve as an opportunity for educating clinical trial support staff at the study site as well as those at other sites. However, further study is warranted to determine the role of the clinical trial support staff in optimizing methods for managing adverse events requiring compensation in registration trials.

## Background

Clinical research plays an important role in improving the quality of medical practice. In particular, registration trials leading to the approval of drugs and medical devices are paramount in the development of drugs and medical devices. In 1996, the Good Clinical Practice (GCP) was introduced as a global standard, and since the Japanese introduction of it in 1997, the infrastructure for registration trials in Japan has improved. The contribution of clinical trial support staff, such as clinical research coordinators (CRC) and administrative officers, is now widely recognized.

CRC must engage not only in the scientific and ethical pursuit of registration trials, but also in assuring standards of quality by coordinating the activities and conduct of participants, investigators, sponsors, and various hospital staff. Among these duties, adverse event management, including proper and timely reporting to sponsors, is a key responsibility. CRC, as well as investigators, should pay close attention to serious adverse events, partly because these events may lead to compensation events which require additional resources and the involvement of administrative officers. However, on an individual basis, the clinical trial support staff may have limited experience dealing with serious adverse events and compensation. Therefore, reviewing the shared experience of the CRC may offer a valuable opportunity for collecting and analyzing data on the frequency of these events, and provide some insight into appropriate methods for managing them. Accordingly, we retrospectively examined the incidence of serious adverse events and related compensation at Tokushima University Hospital. We present herein the results of our analysis.

## Methods

We retrospectively reviewed records from the institutional review board (IRB) of Tokushima University Hospital for the period 2007 through 2011. A serious adverse event was defined by the criteria established in the International Conference on Harmonisation (ICH) harmonised tripartite guideline E2A [1]. We classified a serious adverse event as an untoward medical occurrence which at any dose: 1) resulted in death, 2) was life-threatening, 3) required inpatient hospitalisation or prolonged an existing hospitalisation, 4) resulted in persistent or significant disability or incapacity, 5) was a congenital anomaly/birth defect, or 6) others. We compared the frequency of events and subjects stratified by malignant and non-malignant diseases and applied a chi-squared test to analyze differences. A two-sided *P*-value <0.05 was considered to be statistically significant. This study was approved by the Ethics Committee of Tokushima University Hospital.

## Results

### Number of registration trials and subjects over the five-year period

Table 1 presents the number of registration trials approved by the IRB at Tokushima University Hospital each year as well as the frequency of subjects with malignant diseases and non-malignant diseases. Data for the year 2007 pertained to trials continuing from previous years as well as those approved in the current year. For 2008–2011, clinical trials approved in each respective year were included. As shown in Table 1, clinical studies in non-malignant diseases comprised a greater proportion of registration trials conducted at Tokushima University Hospital compared to those in malignant diseases.

**Table 1:** Frequency of studies and subjects in oncology and non-oncology registration trials at Tokushima University Hospital: 2007 to 2011

	**Number of registration trials (%)**			**Number of subjects (%)**		
	**Malignant diseases**	**Non- malignant diseases**	**Total**	**Malignant diseases**	**Non- malignant diseases**	**Total**
2007	11 (23.4)	36 (76.6)	47 (100)	28 (20.7)	107 (79.3)	135 (100)
2008	1 (6.7)	14 (93.3)	15 (100)	5 (7.7)	60 (92.3)	65 (100)
2009	4 (28.6)	10 (71.4)	14 (100)	8 (24.2)	25 (75.8)	33 (100)
2010	4 (28.6)	10 (71.4)	14 (100)	10 (31.3)	22 (68.7)	32 (100)
2011	5 (31.3)	11 (68.7)	16 (100)	2 (10.5)	17 (89.5)	19 (100)
Total	25 (23.6)	81 (76.4)	106 (100)	53 (18.7)	231 (81.3)	284 (100)

### Frequency of serious adverse events

As indicated in Table 2, a total of 43 serious adverse events were reported during the five-year period. Among these, a relationship between the adverse event and investigational drug was ruled out in 14 (66.6%) of 21 events occurring in patients with malignant diseases and in 21 of 22 (95.5%) events in those with non-malignant diseases. Eleven of 21 (52.4%) events in patients with malignant diseases and 6 of 22 (27.3%) events in those with non-malignant diseases led to the withdrawal of the study drug. Examining the numbers of subjects with severe adverse events, a significantly greater proportion of subjects with malignant disease (28.3%; 15 of 53) experienced serious adverse events compared to those with non-malignant disease (8.2%; 19 of 231; *P* < 0.01).

**Table 2:** Annual and total number of serious adverse events in subjects with malignant diseases and non-malignant diseases

	**Number of serious adverse events**		
	**Malignant diseases**	**Non- malignant diseases**	**Total**
2007	5	3	8
2008	4	3	7
2009	6	3	9
2010	2	9	11
2011	4	4	8
Total	21	22	43

### Subjects with multiple serious adverse events

Among the 25 oncology registration trials, two subjects experienced two serious adverse events, and two subjects experienced three. None of the subjects in the oncology trials experienced more than three. Among the 81 non-oncology registration trials, one subject experienced two serious adverse events, and another had three. Similar to the oncology trials, none of the subjects in the non-oncology trials had more than three (Table 3).

**Table 3:** Frequency of serious adverse events in subjects with malignant diseases and non-malignant diseases

**Frequency of SAE**	**Number of subjects**		
	**Malignant diseases**	**Non- malignant diseases**	**Total**
0	38	212	250
1	11	17	28
2	2	1	3
3	2	1	3
Total	53	231	284

### Frequency of compensation events and subjects seeking compensation

Over the five-year period, eight compensation events were reported in a total of seven subjects at Tokushima University Hospital. Among these, seven compensation events occurred during the oncology registration trials, and one was associated with a non-oncology trial.

## Discussion

Among the various roles assigned to the CRC, adverse event management, including proper and timely reporting to sponsors, is a key responsibility. The incidence of serious adverse events and compensation may differ across study sites depending on the types of registration trials, investigational drugs, and underlying diseases prevalent in the study population. Among the published studies of Japanese university hospitals, Fukutomi et al. [2] cited 42 serious adverse events and 14 compensation events in 1085 participants over 4.25 years. Yamada et al. [3] reported a total of 78 serious adverse events occurring in 725 participants over three years, whereas Suzuki et al. [4] documented 10 compensation events during a 10-year period. In the present study, 43 serious adverse events and 8 compensation events were reported in 284 subjects over five years, the occurrence of which we consider to be in line with previous reports.

Adverse event management, including proper and timely reporting to sponsors, is an important responsibility of the CRC [5]. A key function of managing serious adverse events is the collection of data on whether or not the subject sought consultation from other medical institutions. Yamada et al. [3] described the development of a manual in an urban university hospital to facilitate the collection of data from physicians in other hospitals with whom subjects consulted for managing serious adverse events during clinical trials. In our rural area, communication between hospitals is readily facilitated, thereby warranting efforts to collect information from other institutions.

Compensation for injured and deceased research subjects has been a long-standing issue [6]. In Japan, sponsors of registration trials are required to prepare for the provision of compensation. Details of compensation are not defined by regulation. Instead, the guideline by the Japan Pharmaceutical Industry Legal Affairs Association [7] is used by most of the sponsors. Japan has universal health coverage by employee-based and community-based social health insurance [8]. In the system, co-payment rate is 30% in general, and the rest is paid by public insurance. Acting of co-payment by sponsors is considered as compensation in Japan. In serious cases, such as fatal cases, monetary compensation is considered.

Because compensation is considered regardless of fault even when the relationship between the adverse event and investigational drug is indeterminate, the investigator’s decision regarding the potential association is highly important. In the present study, a relationship could not be ruled out in 8 of the 43 serious events. Participants’ consent for compensation is mandatory for registration trials. Nevertheless, in most cases, staffs of medical institution propose claims for compensation to injured participants practically. In such cases, the CRC should coordinate the efforts of sponsors, study participants, investigators, and administrative offices from all institutions with the aim of protecting the subjects’ rights, and that is the first role of CRC in compensation. In 2005, we developed a manual to manage compensation events following discussions with the administrative office. In general, it takes time to decide relationship between the adverse event and investigational drug, and participants should pay their co-payment before the decision. As a practice in Tokushima University Hospital, patients’ payment of co-payment is postponed until the final decision of the relationship. Even if payment of co-payment by sponsors starts, it will be ceased when the condition becomes stable. Long time follow-up of participants is also important and these points are shown in our manual. Suzuki et al. [4] also compiled a checklist to manage compensation at a Japanese university hospital. Since individual CRC may seldom participate in compensation events involving their assigned subjects, it may be helpful for those who have dealt with such events to share their experiences with other CRC at the study site as well as with those from other sites.

In the present study, subjects with malignant disease experienced serious adverse events more often than those with non-malignant disease. Conversely, the relationship with the investigational drug was ruled out more frequently in cases with non-malignant diseases than in those with malignant diseases. These findings suggest that the difference was not related to the higher incidence of disease-related death among oncology trials. In a Spanish survey of CRCs attending an educational course on oncology clinical trials, Rico-Villademoros et al. [9] noted that all standard tasks performed by CRCs were categorized as "monitoring activities," and included patient registration/randomization, recruitment follow-up, case report form completion, collaboration with the clinical research associates (CRA), serious adverse events reporting, handling of investigator files, and preparing the site for audits, and attending audits. Although the basic tasks of CRC do not differ, the present findings indicate that CRC should pay close attention to the serious adverse events in oncology clinical trials due to the potentially higher frequency of serious complications in these types of studies.

## Conclusions

The present study evaluated serious adverse events and compensation in registration trials restricted to a university hospital in rural Japan. We found that subjects with malignant diseases experienced serious adverse events and required compensation more frequently than those with non-malignant diseases. From the perspective of the clinical trial support staff, it is necessary to establish more effective systems for managing serious adverse events and compensation. However, given the limitations of our retrospective study, further research is warranted to determine the role of clinical trial support staff in developing a suitable infrastructure for these systems in clinical research.

## Competing interests

The authors declare that they have no competing interests in relation to this article.

## Authors’ contributions

MW, ST and HY conceived the study, collected and analyzed the data, and drafted the manuscript. RK, TM, and MY participated in the study design and collection of data. KM participated in the study design and assisted in drafting the manuscript. All authors have read and approved the final manuscript.

## References

[B1] International Conference on Harmonisation of Technical Requirements for Registration of Pharmaceuticals for Human Use ICH Harmonised Tripartite Guideline. Clinical safety data management: Definitions and standards for expedited reporting E2A [http://www.ich.org/fileadmin/Public_Web_Site/ICH_Products/Guidelines/Efficacy/E2A/Step4/E2A_Guideline.pdf]

[B2] Fukutomi J Terada J Koike N Ishii K Kimura K Kobayashi S St. Marianna Hospital ni okeru yuugaijishou heno taiou to hoshou. [Management of adverse events and compensation in St. Marianna University School of Medicine Hospital] Jpn J Clin Pharmacol Ther 2002 33 53 59 10.3999/jscpt.33.2_53

[B3] Yamada N Hirayama R Matsuura K Onda A Kawai M Ishizaki S Nagamatsu A Watanabe K Toda C Ogura K Arakawa Y Omata M A manual to facilitate clinical data collection from the doctors in other hospitals whom subjects consult for serious adverse events during clinical trials Jpn J Clin Pharmacol Ther 2008 39 111 115 10.3999/jscpt.39.111

[B4] Suzuki R Iwasaki Y Yamamoto Y Asano K Hoshou taiouniokeru CRC you checklist no sakusei to sono unnyou ni tuite [Development and application of CRC checklist for management of compensation] Clin Res Professionals 2012 28 30 33

[B5] Poston RD Buescher CR The essential role of the clinical research nurse (CRN) Urol Nurs 2010 30 1 55 63,77 20359145 10.7257/1053-816x.2010.30.1.55

[B6] Steinbrook R Compensation for injured research subjects N Engl J Med 2006 354 1871 1873 10.1056/NEJMp068080 16672697

[B7] Japan Pharmaceutical Industry Legal Affairs Association Compensation guideline for injured research subjects [http://www.ihoken.or.jp/guideline/2_revisionguidline.pdf]

[B8] Ikegami N Yoo BK Hashimoto H Matsumoto M Ogata H Babazono A Watanabe R Shibuya K Yang BM Reich MR Kobayashi Y Japanese universal health coverage: evolution, achievements, and challenges Lancet 2011 378 1106 1115 10.1016/S0140-6736(11)60828-3 21885107

[B9] Rico-Villademoros F Hernando T Sanz JL López-Alonso A Salamanca O Camps C Rosell R The role of the clinical research coordinator – data manager – in oncology clinical trials BMC Med Res Meth 2004 4 6 10.1186/1471-2288-4-6 PMC406503 15043760

